# Inaccurate identification of rotavirus genotype G9 as genotype G3 strains due to primer mismatch

**DOI:** 10.1186/1743-422X-9-144

**Published:** 2012-08-03

**Authors:** Marcelo Takahiro Mitui, TGA Nilmini Chandrasena, Paul KS Chan, Shaman Rajindrajith, E Anthony S Nelson, Ting Fan Leung, Akira Nishizono, Kamruddin Ahmed

**Affiliations:** 1 Department of Microbiology, School of Medicine, Oita University, Yufu, Oita, Japan; 2 Department of Parasitology, Faculty of Medicine, University of Kelaniya, Ragama, Sri Lanka; 3 Department of Microbiology, Faculty of Medicine, The Chinese University of Hong Kong, Hong Kong, People’s Republic of China; 4 Department of Pediatrics, Faculty of Medicine, University of Kelaniya, Ragama, Sri Lanka; 5 Department of Paediatrics, Faculty of Medicine, The Chinese University of Hong Kong, Hong Kong, People’s Republic of China; 6 Research Promotion Institute, Oita University, Yufu, Oita, Japan

**Keywords:** Group a rotavirus, Genotyping, Primer mismatch, Genotype G3, Genotype G9, Hong Kong, Sri Lanka

## Abstract

Reverse transcription (RT)-PCR is now the standard method for typing group A rotaviruses (RVA) to monitor the circulating genotypes in a population. Selection of primers that can accurately type the circulating genotypes is crucial in the context of vaccine introduction and correctly interpreting the impact of vaccination on strain distribution. To our knowledge this study is the first report from Asia of misidentification of genotype G9 as G3 due to a primer-template mismatch. We tested two published G-genotype specific primers sets, designed by Gouvea and colleagues (Set A) and Iturriza‐Gomara and colleagues (Set B) on RVA from Hong Kong and Sri Lanka. Among 52 rotaviruses typed as G3 by set A primers, 36 (69.2%) were identified as G9 by nucleotide sequencing and set B primers. Moreover, of 300 rotaviruses tested, 28.3% were untypable by set A primers whereas only 12.3% were untypable by set B primers. Our findings reinforce the need to periodically monitor the primers used for RVA genotyping.

## Findings

Every year rotavirus infects 114 million children and accounts for about 453,000 deaths mainly in developing countries [1,2]. RVA is a non-enveloped virus with a triple-layered wheel-like capsid containing 11 segments of double stranded RNA in its core. VP7 and VP4 nucleotide sequence define the G and P genotypes, respectively [3]. These proteins have independent neutralization properties and are used in a binary classification system. Of the 27 G and 35 P types thus far identified by molecular characterization [4], 12 G and 15 P types have been detected in humans [3]. Globally, G1 through G4 and G9 are the most common types causing human infection [5]. The primary goal of most RVA strain surveillance has been to determine the circulating genotypes for the introduction of vaccine and to monitor vaccine effectiveness [6].

RT-PCR is the method of choice for typing rotaviruses and is regarded as the gold standard [7]. The accumulation of point mutations at primer binding sites has been linked with mistyping or failure to identify genotype correctly. To maintain the accuracy, primers used in PCR-based typing methods need to be regularly revised and updated [8]. In 1990, a multiplexed hemi-nested RT-PCR based G genotyping assay was developed by Gouvea and colleagues [9]. This assay, correlated well with an antibody-based G-serotyping system. Subsequently, primers proposed by Das et al. [10] and Iturriza-Gómara et al. [11] for G genotyping assays were introduced.

In our laboratory we used primers designed by Gouvea et al., for genotyping RVA from different countries and satisfactory results were obtained when RT-PCR results were compared with VP7 gene sequence information. We regularly validated the results of PCR with VP7 sequence results and, during genotyping of RVA from Sri Lankan samples, it was identified that many of the genotype G9 rotaviruses were typed as G3 by PCR.

G9 strains started to be reported in the mid-1990s from India, Japan, the United Kingdom, and the United States [12]. Subsequently, these strains spread globally and they are now the third most common genotype of RVA. In recent years the G9 rotaviruses have also shown increasing genomic diversity within the genotype [13].

An overall increase in G3 strains has recently being reported from many countries [14-16], some of them in association with the introduction of the pentavalent RVA vaccine [17,18]. How vaccine pressure has resulted in emergence of this genotype is unknown, but sustained and accurate monitoring is clearly desirable to clarify this.

Since both G9 and G3 are very important considering the current epidemiology of genotype distribution, we extended the experiment to all samples from Sri Lanka and Hong Kong and determined that alternate primers set designed by Iturriza-Gomara et al. (2004) [11] gave us satisfactory results. Considering the importance of this finding we are reporting the results to aware researchers.

A total of 422 stool samples positive for RVA were obtained from children under 5 years of age who attended major hospitals in Hong Kong and Sri Lanka for diarrhea: 300 from Hong Kong (December 2004 through December 2005); 122 from Sri Lanka (April 2005 through October 2006). These samples were collected as part of rotavirus surveillance [14,19]. Approval was obtained from the Ethical Review Board of Sri Lankan College of Pediatrics. Hong Kong samples were the remaining portion of specimens submitted for tests because of clinical indication, therefore approval was not required. All samples were delinked from personal identifiers.

RVA was detected using the commercially available ELISA kits according to manufacturer’s instructions: IDEIA Rotavirus (Dako Diagnostics, Cambridgeshire, UK) in Hong Kong; and Rotaclone (Meridian Bioscience Inc., Cincinnati, OH) in Sri Lanka. The nucleic acids were extracted by the phenol- chloroform-isoamyl alcohol method.

We used one step RT-PCR targeting the VP7 gene of RVA. PCR primers were Beg9 and End9 [9]. A commercial master mix formulation, AccessQuick™ RT-PCR System (Promega Corporation, Madison, WI, USA) was employed. Each reaction contained 25 μl of AccessQuick™ Master Mix, 1 μl of each primer at a final concentration of 0.2 μM, 2 μl of the dsRNA template, 1 μl of reverse transcriptase, and nuclease-free water to a final volume of 50 μl. For RT, incubation was at 45°C for 45 minutes and the contiguous PCR conditions were as follows: 95°C for 1 minute, followed by 40 cycles at 94°C for 1 minute, 50°C for 1 minute, 72°C for 1 minute 30 seconds and a final extension at 72°C for 5 minutes.

In this study, the G type was identified by hemi-nested multiplex PCR method with genotype specific primers for G1, G2, G3, and G4 proposed by Gouvea et al. [9] (designated “set A”) and by nested multiplex PCR method with genotype specific primers for G1, G2, G3, G4 and G9 proposed by Iturriza-Gómara et al. [11] (designated “set B”)(Table 1). For both sets of primers, PCR was carried out by the PCR Master Mix (Promega Corporation), as follows: each reaction contained 25 μl of PCR Master Mix, 1 μl of each primer at a final concentration of 0.2 μM, 1 μl of the VP7 amplicon, and nuclease-free water to a final volume of 50 μl. PCR conditions were as follows: 30 cycles at 95°C for 1 minute, 42°C for 2 minutes and 72°C for 1 minutewith a final extension step at 72°C for 5 minutes.

**Table 1:** Primer comparison between Gouvea et al. and Iturriza-Gomara et al. Note that primer for G3 and G9 are different

**Primers of Gouvea et al. (Set A)**		**Primers of Iturriza-Gomara et al. (Set B)**	
**Primer name**	**Primer sequence**	**Primer name**	**Primer sequence**
aBT1	CAAGTACTCAAATCAATGATGG	G1	CAAGTACTCAAATCAATGATGG
aCT2	CAATGATATTAACACATTTTCTGTG	G2	CAATGATATTAACACATTTTCTGTG
aET3	CGTTTGAAGAAGTTGCAAG	G3	ACGAACTCAACACGAGAG
aDT4	CGTTTCTGGTGAGGAGTTG	G4	CGTTTCTGGTGAGGGTTG
aFT9	CTAGATGTAACTACAACTC	G9	CTTGATGTGACTAYAAATAC

Primers for G1, G2 and G4 in Iturriza-Gomara et al. were adapted from primers of Gouvea et al.

The nucleotide sequence of the VP7 gene was determined by Big Dye terminator v3.1 cycle sequencing kit (Applied Biosystems, Foster city, CA, USA) and the product was subjected to an ABI Prism 3130 Genetic Analyzer (Applied Biosystems) [12]. The VP7 gene sequence similarity was searched by BLAST to determine the G types. The genotype of each strain determined by different sets of primers was compared with corresponding nucleotide sequence.

Of the 52 RVA typed as G9 by nucleotide sequencing of the VP7 gene, 36 (69.2%) (3 from Hong Kong and 33 from Sri Lanka) were typed as G3 by set A primers.

**Figure 1:**
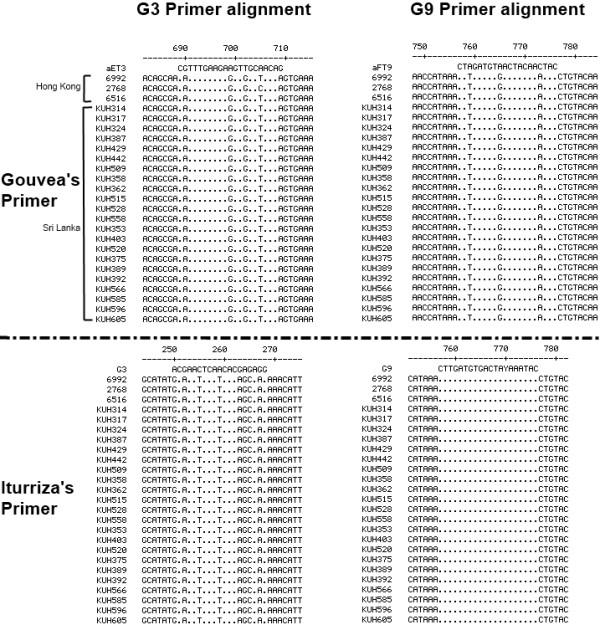
Multiple alignments of the VP7 gene sequences of G9 rotaviruses from Hong Kong and Sri Lanka with the corresponding G3 and G9 specific primers proposed by Gouvea et al. and Iturriza-Gómara et al. Dots indicate consensus with the primer

To determine the identity between the primers and the nucleotide sequences of the gene, the G3 and G9 primers of both set A and B were aligned with the nucleotide sequence of the VP7 gene of G9 strains (3 from Hong Kong and 22 from Sri Lanka) (Figure 1). The G3 and G9 primers of set A showed 76% (16/21nt) and 85% (17/20nt) identity, whereas the G3 and G9 primers of set B had 58% (11/19nt) and 100% (20/20) identity, respectively.

Aligning the G3 primers of set A with the VP7 gene sequences of G9 strains revealed eight consecutive nucleotide identities in the middle and three consecutive nucleotide identities next to the 3′ end. This could explain the mismatched primer binding and as a consequence the incorrect genotyping.

G3 forward primer of set A was derived from residues 689 to 709 nt of the VP7 gene of G3 RVA generating a PCR product of 374 bp, but it also anneals with the same residues of the VP7 gene of G9 RVA, generating a PCR product of the same length and indistinguishable after running the gel electrophoresis. G3 forward primer of set B was derived from residues 250 to 269 nt of the VP7 gene of G3 rotavirus generating a PCR product of 682 bp.

To verify the accuracy of set B primers, typing was done on five G9 and five G3 strains. The types of these strains were confirmed by nucleotide sequencing. All were typed correctly by set B primers but identified as genotype G3 by set A primers.

A total of 300 rotaviruses arbitrarily selected from Hong Kong were subjected to typing by both sets of primers, 80 strains were typed as G3 by both sets of primers. However, nine G9 strains typed as G3 by set A primers were correctly typed by set B primers, showing a 10.1% chance of mistyping (G9 as G3) by set A primers. Of 300 rotaviruses tested, 28.3% were untypable by set A primers whereas only 12.3% were untypable by set B primers. These untypables were typed by the VP7 gene sequencing and no primer mismatch was found indicating possible contaminants which hindered PCR.

RT-PCR genotyping failure is a hindrance in RVA research, generally ranging 10–30%, and tends to be higher in samples from developing countries [20]. This problem has often been attributed to single nucleotide polymorphisms at the primer binding sites [21-25]. The occurrence of erroneous typing among strains due to mismatched primers deserves special consideration. Reported RVA primers mismatches identified genotype G8 as G12 and G3 as G10 [25-27]. To our knowledge this is the first report from Asia of G9 being mistyped as G3 [28], since both are prevalent genotypes worldwide, this information will be crucial for accurate surveillances.

Gouvea et al. [9] and Iturriza-Gómara et al. [11] proposed the primers for G genotyping in 1990 and 2004, respectively. It is plausible that rotaviruses have high mutation rates even in sites that are considered well conserved. It is therefore essential that widely used primers sets, particularly those developed some while back, be re-evaluated on a regular basis to ensure that circulating RVA strains are accurately typed.

## Competing interests

P. K. S. Chan participated in vaccine studies funded by Baxter, GlaxoSmithKline and MedImmune, and received travel support from GlaxoSmithKline, Merck and Roche. E. A. S. Nelson has received funding from Merck and Pfizer (Wyeth) for diarrhoeal and respiratory disease surveillance studies, has participated in vaccine studies funded by Baxter, GlaxoSmithKline, MedImmune and Wyeth, including a Phase III Rotarix study, and has received lecture fees and travel support from GlaxoSmithKline, Merck, Intercell and Pfizer (Wyeth). Other authors have no conflict of interest.

## Authors’ contributions

Conceived and designed the experiments: MTM, KA. Performed the experiments: MTM, TGANC, CPKS, SR, TFL. Analyzed the data: MTM, KA, AN. Wrote the paper: MTM, KA, CPKS, EASN. All the authors have read and approved the final manuscript.
